# Working conditions and health behavior as causes of educational inequalities in self-rated health: an inverse odds weighting approach

**DOI:** 10.5271/sjweh.3918

**Published:** 2021-03-01

**Authors:** Jolinda LD Schram, Joost Oude Groeniger, Merel Schuring, Karin I Proper, Sandra H van Oostrom, Suzan JW Robroek, Alex Burdorf

**Affiliations:** Department of Public Health, Erasmus Medical Centre Rotterdam, The Netherlands; Department of Public Administration and Sociology, Erasmus University, Rotterdam, The Netherlands; Centre for Nutrition, Prevention and Health Services, National Institute for Public Health and the Environment (RIVM), Bilthoven, The Netherlands

**Keywords:** causal mediation, longitudinal analysis, mediation analysis, socioeconomic inequality

## Abstract

**Objective:**

Using a novel mediation method that presents unbiased results even in the presence of exposure–mediator interactions, this study estimated the extent to which working conditions and health behaviors contribute to educational inequalities in self-rated health in the workforce.

**Methods:**

Respondents of the longitudinal Survey of Health, Ageing, and Retirement in Europe (SHARE) in 16 countries were selected, aged 50–64 years, in paid employment at baseline and with information on education and self-rated health (N=15 028). Education, health behaviors [including body mass index (BMI)] and working conditions were measured at baseline and self-rated health at baseline and two-year follow-up. Causal mediation analysis with inverse odds weighting was used to estimate the total effect of education on self-rated health, decomposed into a natural direct effect (NDE) and natural indirect effect (NIE).

**Results:**

Lower educated workers were more likely to perceive their health as poor than higher educated workers [relative risk (RR) 1.48, 95% confidence interval (CI) 1.37–1.60]. They were also more likely to have unfavorable working conditions and unhealthy behaviors, except for alcohol consumption. When all working conditions were included, the remaining NDE was RR 1.30 (95% CI 1.15–1.44). When BMI and health behaviors were included, the remaining NDE was RR 1.40 (95% CI 1.27–1.54). Working conditions explained 38% and health behaviors and BMI explained 16% of educational inequalities in health. Including all mediators explained 64% of educational inequalities in self-rated health.

**Conclusions:**

Working conditions and health behaviors explain over half of the educational inequalities in self-rated health. To reduce health inequalities, improving working conditions seems to be more important than introducing health promotion programs in the workforce.

Reducing educational inequalities in health is one of the main challenges for public health (1, 2). Various studies have identified potential determinants of educational inequalities in morbidity and mortality by examining mediating factors that explain the association between education and health (1, 3, 4). Many explanations have been presented, ranging from behavioral risk factors and subjective economic status to material and occupational factors (1, 5–7).

People with a low educational level have a poorer self-rated health than those with a high educational level. In the European Union, 56% of people with low education were in very good or good self-rated health, compared to 80% of the high educated in 2018 (8). Poor working conditions and unhealthy behaviors are more prevalent in lower compared to higher socioeconomic groups (9–11) and are associated with poor health (12–14). Previous studies have estimated that these factors may explain approximately two thirds of the association between socioeconomic position and self-rated health (13). A recent review summarized that work factors explained about one third of socioeconomic inequalities in self-rated health and health behavior about one fifth (6).

To determine to what extent risk factors contribute to educational inequalities in health, studies have mainly used traditional approaches to mediation analysis, primarily the so-called ‘difference method’ (15–17). This method assesses mediation by estimating the reduction in the excess (health) risk of lower educational groups compared to the highest educational group after conditioning on the mediator(s). This approach, however, is only valid in linear models under the assumption that there is no interaction between the exposure and mediator on the outcome (18, 19). Several studies have shown that this crucial assumption is often violated (18), also in studies on socioeconomic inequalities in health (15, 20, 21).

Counterfactual or causal mediation analysis bypass the need to rely on this assumption but are still rarely utilized in observational studies (22). This may be related to a lack of flexibility of current causal mediation methods, such as limitations to include multiple mediators (23). Recently, Tchetgen introduced the inverse odds weighting (IOW) approach to estimate natural direct and indirect effects (NDE and NIE), which accommodates effect decomposition with multiple mediators (regardless of their scale), even in the presence of exposure–mediator interactions and nonlinearities (24). Because it is likely that the effect of an unhealthy lifestyle and working conditions on health is different for low compared to high educated persons (3, 25, 26), this study utilizes the IOW approach to estimate to what extent educational inequalities in self-rated health are mediated by working conditions and health behavior.

## Methods

### Data

The study population consisted of participants of the Survey of Health, Ageing, and Retirement in Europe (SHARE),a longitudinal study that collects health, social, and economic data on the population aged ≥50 years every two years. It started in 2004 and 2005 in 11 European countries (Sweden, Denmark, the Netherlands, Belgium, Germany, Austria, Switzerland, France, Italy, Spain and Greece) (27). In 2006, the Czech Republic, Ireland and Poland joined and, in 2010, Estonia and Slovenia. The SHARE sampling design varied in the participating countries, ranging from random selection of households to multistage designs, due to various institutional settings. Details on data collection, comparability of data, and response levels are provided by the official SHARE documentation found at www.share-project.org/data-documentation.html.

For this study, given different periods of enrolment in the SHARE study, respondents who participated in at least two consecutive waves were selected if they were aged 50–64 and employed at the first wave and had information on self-rated health in the subsequent wave. The upper age range was chosen to focus on age groups with substantial participation in paid employment. Five waves were used for the analysis and the total sample included ­ 15 028 respondents. For a division per country and wave, see the supplementary material (www.sjweh.fi/show_abstract.php?abstract_id=3918) table S1. All data were self-reported.

### Self-rated health

The outcome of this study was self-rated health for which a single item question was used. Respondents could indicate whether their health was excellent, very good, good, fair or poor. Self-rated health was dichotomized into ‘less than good’ (poor or fair) and ‘good ’ (good, very good or excellent) (28).

### Educational level

Highest level of education was coded according to the 1997 International Standard Classification of Education (ISCED-97) and categorized into low (0–2: pre-primary, primary and lower secondary education), intermediate (3–4: upper secondary education/post-secondary non-tertiary) and high (5–6: tertiary education).

### Body mass index (BMI) and health behaviors

BMI was calculated by dividing body weight in kilogram by the square of body height in meters. BMI was categorized into normal weight (<25 kg/m^2^), overweight (≥25–<30 kg/m^2^), and obese (≥30 kg/m^2^). Smoking status was measured with two questions and categorized into three categories: non-, former, and current smoker (27). Alcohol consumption was based on the number of days per week participants drank alcohol during the last three months (in wave 1 during the last six months): <1 day, 1–2 days (reference category), 3–4 days, 5 days per week (29). In our analyses, BMI is included in health behaviors.

### Working conditions

Three variables were included for working conditions: physical job demands, job control, and job rewards. All working conditions were assessed by items derived from the Job Content Questionnaire on the demand–control model (30) and the effort–reward imbalance model questionnaire (31). Physical job demands were measured using a single question: “My job is physically demanding”. Individuals who indicated strongly agree or agree were considered to have a physically demanding job. Job control and rewards were included in this study as underlying dimensions of the job demand–control model and effort–reward imbalance since previous research has shown that these factors are more prevalent among workers with a low compared to high educational level, while high job demands are more prevalent among workers with a high educational level (10). Moreover, previous research also showed that low rewards and low job control are the main drivers in the association between job demand–control or effort–reward imbalance and exit from paid employment (32). Job control was measured by using the sum score of two items: (i) “I have very little freedom to decide how I do my work” and (ii) “I have an opportunity to develop new skills”. Questions were recoded to ensure that higher values indicate higher physical job demands and lack of job control. Rewards were measured by using the sum score of five items addressing support, recognition, salary/earnings, job promotion prospects, and job security. All items were measured on a 4-point scale ranging from 1=strongly agree to 4=strongly disagree. Questions were recoded to ensure that higher values indicate a lack of rewards.

Following Dragano et al (33), we used the upper country-specific tertile of the scale distribution for lack of rewards and lack of job control. As previous analyses showed that the measures of job control and rewards varied across countries, tertiles were calculated for each country separately (34).

### Covariates

Cohabitation was used to categorize individuals into living with a spouse or partner and living alone. Sex was a dichotomous covariate, while age was a categorical variable, divided into three categories 50–54, 55–59 and 60–64 years. Self-rated health at baseline, country and wave were also covariates.

### Statistical analysis

Education, health behaviors and working conditions were measured at the first available wave, while self-rated health was measured at baseline and the consecutive wave at two-year follow-up. Since the study population is ≥50 years, we can reasonably assume that educational attainment has preceded working conditions and health behavior, even though they were measured at the same wave. Missing variables (ranging 0– 3.48%, table 1) were imputed using multiple imputation by chained equations (M=20) (35). Descriptive statistics were used to describe the prevalence of the health behaviors and working conditions in each educational category.

**Table 1:** Sample characteristics among imputed dataset of employed individuals at baseline (N=15 028). [t=time.]

	Educational level			% imputed

	Low(N=3837)	Intermediate (N=5599)	High (N=5592)	

	%	%	%	
Women	46	47	51	
Age (years)				
50–54	47	54	49	
55–59	37	34	35	
60–64	16	12	16	
Not cohabitating	20	23	23	2.38
Self-perceived health (t0)				
Less than good	22	19	14	0.06
Self-perceived health (t1)				
Less than good	26	23	16	
Health behaviors				
Body mass index				
Normal	35	40	48	3.48
Overweight	45	42	38	
Obese	21	18	13	
Smoking				
No	40	43	48	0.03
Current	32	28	21	
Former	27	29	31	
Alcohol consumption				
Hardly ever/never	33	28	22	0.05
1–2 days per week	37	41	43	
3–4 days per week	7	10	13	
5 or more days per week	23	21	22	
Working conditions				
Physically demanding job	65	49	32	0.59
Lack of job control	41	36	22	0.56
Lack of job rewards	37	35	28	0.53

**Figure 1:**
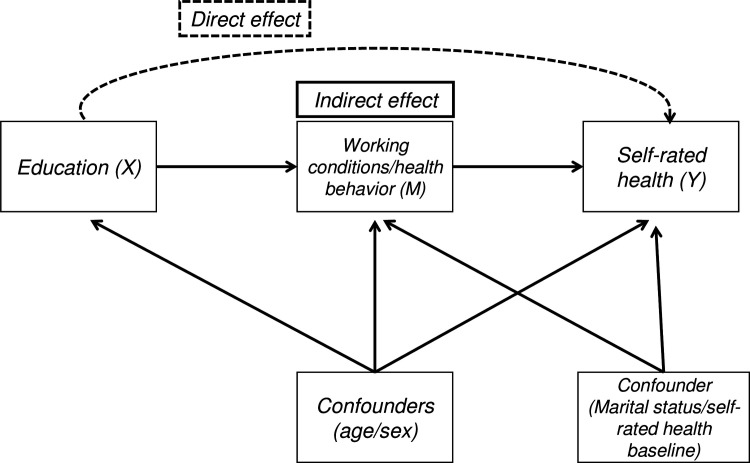
Representation of the hypothesized relationship between education, working conditions/health behavior and self-rated health.

Due to significant interactions between education, job control and job rewards, causal mediation analysis was used to estimate the NDE, NIE, and total effect (TE). The TE of education on self-rated health (comparing low versus high educated and low versus mid educated) was decomposed into the effect occurring through the mediators of interests (NIE) – ie, working conditions and health behaviors – and the effect occurring through other pathways (NDE) (figure 1). The NIE estimates the expected change in self-rated health among low educated persons, if, potentially counter to the fact, the mediators were changed from the level of the low educated to the level of the high educated, with educational level being fixed at low education. The NDE expresses the expected change in educational inequalities in self-rated health if, potentially counter to fact, the individual would change from being low to high educated, but the mediators of interest were kept at the level they would have taken for the high educated (ie, the referent level) (19).

In order to identify the NIE and NDE, four assumptions have to be made: no unmeasured confounding between (i) the exposure and the mediator, (ii) the mediator and the outcome and (iii) the exposure and outcome. Furthermore, (iv) there are no confounders of the mediator–outcome relationship that are itself affected by the exposure (36). The NDE, NIE, and TE were calculated using the IOW approach. In this approach the mediator itself is not entered into the regression model for the outcome but is only used in the construction of a weight. By applying this weight in the outcome model, the exposure and mediator are effectively independent and the indirect pathway involving the mediator is deactivated (16).

Following Tchetgen Tchetgen (24) and Nguyen et al (16), the mediation analysis consisted of five steps. First, a multinomial logistic model was fitted regressing the exposure (education) on the mediators and covariates. Second, an IOW weight was computed by taking the inverse of the predicted odds from step 1 for each observation. The reference group’s weight, either mid or high educated, was set at 1. Third, the NDE was estimated using generalized linear models with a Poisson distribution and log-link function regressing the outcome on exposure and covariates, weighted by the previously calculated IOW weight. Poisson models were used instead of logistic regression models because for common outcomes the odds ratio is non-collapsible, which underestimates mediation effects (16). Fourth, the TE was estimated using a generalized linear model with a Poisson distribution and log-link function regressing the outcome on exposure and covariates (without including the weights). Fifth, the NIE was calculated by subtracting the NDE from the TE. The effect estimates were bootstrapped (1000 repetitions) to derive confidence intervals (CI) for the NDE, NIE and TE.

The steps for the IOW regression were followed for each mediator separately (tables 2 and 3) and all mediators combined (table 4). Self-rated health at baseline, country, age, sex, cohabitation and wave dummies were included as covariates. Finally, a measure of the ‘‘proportion mediated’’ (PM) on the risk ratio (RR) scale was calculated using the equation provided by VanderWeele (37, 38).

**Table 2:** Total, natural direct and natural indirect effect (NDE and NIE) of education on less than good self-rated health (N=15 028) with health behaviors as mediators. [RR=relative risk; CI=confidence interval]

	Low versus middle socioeconomic position			Low versus high socioeconomic position		
	
	RR	95% CI	Proportion mediated %	RR	95% CI	Proportion mediated %
Body mass index ^a^						
NIE	1.02	0.97–1.06	12	1.00	0.94–1.06	0
NDE	1.15	1.06–1.24		1.48	1.34–1.62	
Alcohol consumption^a^						
NIE	1.02	0.98–1.06	14	1.00	0.95–1.06	1
NDE	1.15	1.05–1.24		1.47	1.34–1.61	
Smoking ^a^						
NIE	1.01	0.97–1.06	9	0.99	0.94–1.04	-3
NDE	1.15	1.06–1.25		1.49	1.36–1.63	
Total effect	1.17	1.09–1.25		1.48	1.37–1.60	

a Adjusted for self-rated health at baseline, cohabitation, age, country, sex and wave.

**Table 3:** Total, natural direct and natural indirect effect (NDE and NIE) of education on less than good self-rated health (N=15 028) with working conditions as mediators. [RR=relative risk; CI=confidence interval]

	Low versus middle socioeconomic position			Low versus high socioeconomic position		
	
	RR	95% CI	Proportion mediated %	RR	95% CI	Proportion mediated %
Physically demanding job ^a^						
NIE	1.06	1.01–1.11	38	1.08	1.00–1.16	24
NDE	1.11	1.02–1.20		1.37	1.23–1.51	
Lack of job control ^a^						
NIE	1.04	0.99–1.08	24	1.06	0.99–1.12	16
NDE	1.13	1.04–1.22		1.40	1.26–1.54	
Lack of rewards ^a^						
NIE	1.01	0.97–1.25	7	0.99	0.94–1.04	–2
NDE	1.16	1.07–1.25		1.49	1.35–1.63	
Total effect	1.17	1.09–1.25		1.48	1.37–1.60	

a Adjusted for self-rated health at baseline, cohabitation, age, country, sex and wave.

**Table 4:** Total, natural direct and natural indirect effect (NDE and NIE) of education on less than good self-rated health (N=15 028) with health behaviors and working conditions as mediators. [RR=relative risk; CI=confidence interval]]

	Low versus middle socioeconomic position			Low versus high socioeconomic position		
	
	RR	95% CI	Proportion mediated %	RR	95% CI	Proportion mediated %
Working conditions ^a^						
NIE	1.08	1.03–1.13	51	1.14	1.04–1.24	38
NDE	1.08	0.99–1.17		1.30	1.15–1.44	
Health behaviors ^a^						
NIE	1.04	0.99–1.08	25	1.05	0.99–1.12	16
NDE	1.13	1.03–1.22		1.40	1.27–1.54	
Health behaviors & working conditions ^a^						
NIE	1.11	1.06–1.17	71	1.26	1.15–1.37	64
DE	1.05	0.96–1.14		1.17	1.04–1.31	
Total effect	1.17	1.09–1.25		1.48	1.37–1.60	

a Adjusted for self-rated health at baseline, cohabitation, age, country, sex and wave

Proportion mediated = RR_NDE_ (RR_NIE_ -1) / (RR_TE_ -1)

All analyses were conducted in Stata V15.1 (Stata Corp, College Station, TX, USA).

### Sensitivity analysis

We conducted sensitivity analyses to check the robustness of our results. First, analyses were repeated not accounting for self-rated health at baseline (supplementary tables S2, S3, and S4). Second, we repeated the mediation analysis using the traditional difference method (supplementary table S5). Third, the analyses including health behaviors and working conditions as mediators were also separately conducted by European region (supplementary tables S6 and S7), since previous research has shown that educational inequalities in health behavior differs substantially across European regions (eg, north-south gradients for smoking among women) (39).

## Results

There was a higher prevalence of less than good health with lower education (table 1). Overweight and obesity were more prevalent among lower educated persons, whereas alcohol use was more common among highly educated persons. Non- and former smokers were more prevalent among higher educated persons. Lower educated persons had poorer working conditions. Physically demanding work and low job control had a stronger social gradient than low job rewards.

Table 2 presents the educational inequalities in self-rated health and impact from health behaviors. Low educated persons reported 1.17-fold (95% CI 1.09–1.25) and 1.48-fold (95% CI 1.37–1.60) higher occurrence of poor self-rated health compared to respectively intermediate and highly educated persons. If low educated persons were to have the BMI or health behaviors of high educated persons, educational inequalities would be still be 1.47–1.49. Table 3 shows the educational inequalities and the impact of unfavorable working conditions. If low educated persons were to have the same physical demanding jobs as high educated, educational inequalities would be reduced to 1.37 (95% CI 1.23–1.51). A similar reduction is shown for lack of job control, while a lack of rewards does not show a reduction in educational inequalities.

If low educated persons were to adopt all health behaviors of high educated persons, educational inequalities in self-rated health would reduce from 1.48 (95% CI 1.37–1.60) to 1.40 (95% CI 1.27–1.54) (table 4). If low educated persons were to have the same working conditions as high educated persons, educational inequalities would reduce from 1.48 to 1.30 (95% CI 1.15–1.44). Furthermore, the estimated reduction in educational inequalities in self-rated health by working conditions was up to 38%, while health behavior explained 16% of the inequalities. Including both working conditions and health behaviors, these factors together explained up to 64% of the educational inequalities in self-rated health.

### Sensitivity analysis

Not adjusting for self-rated health at baseline (supplementary tables S2, S3 and S4), working conditions explained 31% (low versus high education: TE 1.82 to NDE 1.57) to 35% (low versus middle education: TE 1.31 to NDE 1.20) of the educational inequalities in self-rated health, while health behavior explained 27% (low versus high education: TE 1.82 to NDE 1.60; low versus middle education: TE 1.31 to NDE 1.22). Together these factors explained 53–54% of the educational inequalities. In the traditional difference method, the estimated mediation effects of low versus high education were similar for working conditions and for health behavior to the results from the IOW approach, while the combined effect differed substantially from the IOW approach (PM 50% versus 64%) (supplementary table S5). Results from separate analyses per European region (supplementary tables S6 and S7), showed that health behaviors contributed more to educational inequalities in the Northern countries compared to the Southern countries (NIE 1.20 and 1.01, respectively), whereas working conditions contributed more in Southern countries than in Northern countries (NIE 1.32 and 0.98, respectively). Supplementary table S8 showed that educational gradients in health behaviors were more pronounced in Northern Europe than in Southern Europe and educational gradients in poor working conditions were greater in Southern Europe than in Northern Europe.

## Discussion

This study showed that unhealthy behaviors and poor working conditions contribute strongly to educational inequalities in older employees’ self-rated health. While the separate mediators accounted for a small part of the educational inequalities, combined these mediators accounted for 71% of the educational inequalities in self-rated health comparing low to intermediate educated persons and 64% comparing low to high educated persons.

A systematic review of studies assessing mediators between education and self-rated health suggests that material factors, a wider category including working conditions, contribute more to socioeconomic inequalities than behavioral factors (40). A recent review showed that working conditions explained about one third of the socioeconomic inequalities in self-rated health in the working population, while health behavior accounted for about one fifth (6). Although the range and detail of working conditions in our study is limited and the studies differ in years of follow-up, our results seem to align with this review for working conditions, whereas the contribution of health behavior was slightly smaller in our results. The association between poor working conditions and self-rated health at follow-up remained, even when adjusting for self-rated health at baseline (supplementary table S7). Our results align to previous research when accounting for self-rated health at baseline in the sensitivity analyses, which showed a larger contribution of working conditions to educational inequalities in self-rated health, while the contribution of health behavior decreased.

The majority of research on the contribution of working conditions and health behavior to health inequalities has focused on workers in general with limited longitudinal evidence (6). Specific studies focusing on the older population are scarce and focused on either health behavior (41) or working conditions (42). In a German study, using the German SHARE data, behavioral factors explained 19% of the association between low education and self-rated health (41). In an American study, control at work was important for socioeconomic inequalities in self-rated health, while physical demands were not (42). Our study adds to this by analyzing the influence of working conditions and health behavior simultaneously in a longitudinal dataset, showing that working conditions are of more importance than health behaviors in explaining health inequalities among older workers.

To our knowledge, this paper is the first to use causal mediation analysis to assess the relative contribution of working conditions and health behaviors to educational inequalities in self-rated health within the older working population. The traditional difference method is often employed to assess mediation, but it only gives correct direct and indirect effects under very stringent assumptions (24). The advantage of the IOW method is that it can provide direct and indirect effect estimates, even in the presence of nonlinearities and interactions (24). Because the mediator itself is never entered in the outcome model, but only indirectly through the creation of the weights, exposure–mediation interactions do not have to be specified and fewer modelling assumptions are required (43). As shown in the sensitivity analysis, the results of the analyses comparing persons with a low to those with a high educational level in the IOW approach were in line with the traditional method, although in the traditional method the proportion mediated for the low versus middle educated group is underestimated compared to the IOW approach. Which method is most appropriate to use will vary from study to study and relies on evaluating the pros and cons of the different approaches. In our study, IOW was most appropriate due to exposure–mediator interactions found in our dataset.

Our results suggest that working conditions are more important than health behaviors in explaining educational inequalities in self-rated health among older workers in Europe. Regional differences are profound. Sensitivity analyses showed that, in northern countries, educational inequalities in health were more strongly affected by health behavior, while in southern Europe working conditions were more important. This implies that policies and interventions to reduce educational inequalities in health are more promising when they are targeted towards reducing unhealthy behaviors in lower educational groups in northern Europe and targeted towards reducing poor working conditions in lower educational groups in southern Europe.

In our study, a large longitudinal dataset with comparable procedures in data collection and study design across countries was used to test the contribution of working conditions and health behavior to educational inequalities in self-rated health. However, the study has some limitations. First, people with good self-rated health were more likely to participate in the study, as in next waves of SHARE, participants were more likely to be in good health (44). This self-selection process may differ across educational levels, and, thus educational inequalities may have been affected. Second, working conditions or health behaviors at earlier life stages were not accounted for, and these may have affected the educational inequalities in self-rated health due to the combined and cumulative effect of risk factors over the life course (45). Third, interpretation of NIE estimates as causal depends on several unmeasured confounding assumptions underlying causal mediation analysis. Although we adjusted for self-rated health at baseline, results could still be biased due to other unmeasured confounders, such as childhood or adolescent health. Fourth, all data were self-reported. Persons reporting poor self-rated health may also tend to report poor working conditions and poor health behavior, leading to reporting bias. Future studies using not just self-reported indicators are needed to conduct similar analyses. Furthermore, the working conditions reported were limited and relatively crude in comparison to surveys that specifically focus on working conditions. Cross-sectional studies have found a larger contribution of working conditions to educational inequalities in self-rated health when including biomechanical and chemical exposures (6). For self-rated health, research has shown it is strongly predictive for objective health measures, such as mortality (46).

### Concluding remarks

Our findings show that both strenuous working conditions and, to a smaller extent, unhealthy behaviors contribute to educational inequalities in older employees’ self-rated health. This expands the knowledge basis for prevention strategies aiming to reduce socioeconomic inequalities in health.

### Funding

ZonMw funded this study (no. 531001412). The funder had no role in the study design; in the collection, analysis, and interpretation of data; in the writing of the report; or in the decision to submit the manuscript for publication.

### Data sharing statement

The SHARE data are publicly available at www.share-project.org/data-access/user-registration.html. The authors of this manuscript are not authorized to provide data directly to any users.

## Supplementary material

Supplementary material
