# Supplementary material for: Working conditions and health behavior as causes of educational inequalities in self-rated health: an inverse odds weighting approach
Source: Scand J Work Environ Health. 2021 Mar 1;47(2):127–35. doi: 10.5271/sjweh.3918 (PMC8114570; doi:10.5271/sjweh.3918)
Supplement: Supplementary material [file SJWEH-47-127-S001.pdf]

Working conditions and health behavior as causes of educational inequalities in self-rated health: an inverse odds weighting approach [1]

by Jolinda LD Schram, MSc, Joost Oude Groeniger, PhD, Merel Schuring, PhD, Karin I Proper, PhD, Sandra H van Oostrom, PhD, Suzan JW Robroek, PhD,[2] Alex Burdorf, PhD

1. *Supplementary material*
2. *Correspondence to: Suzan J W Robroek, Department of Public Health, Erasmus Medical Centre, Rotterdam 3000 CA, The Netherlands. [E-mail: s.robroek@erasmusmc.nl]*

Supplementary table 1: Participants per country and waves of SHARE

Country	Wave 1 - SRH wave 2	Wave 2 – SRH wave 3	Wave 4 – SRH wave 5	Total
Austria	179	19	785	983
Germany	449	217	21	687
Sweden	828	99	26	953
Netherlands	496	233	274	1003
Spain	271	161	348	780
Italy	286	206	239	731
France	522	241	701	1464
Denmark	427	488	319	1234
Greece	591	242	0	833
Switzerland	259	260	817	1336
Belgium	656	121	740	1517
Czech Republic	0	457	685	1142
Poland	0	297	0	297
Ireland	0	225	0	225
Slovenia	0	0	343	343
Estonia	0	0	1500	1500

Supplementary table 2 Total, natural direct and natural indirect effect of education on less than good self-rated health (n=15,028) for low vs. middle educated group and low vs. high educated group with health behaviors as mediators

	Low versus middle socioeconomic position			Low versus high socioeconomic position		
	Relative Risk	95% CI	Proportion Mediated	Relative Risk	95% CI	Proportion Mediated
**BMI**[1]						
Natural Indirect Effect	1.04	0.99-1.09	16%	1.07	1.01-1.12	14%
Natural Direct Effect	1.26	1.15-1.36		1.71	1.55-1.87	
**Alcohol**[1]						
Natural Indirect Effect	1.04	0.99-1.08	16%	1.06	1.01-1.12	13%
Natural Direct Effect	1.26	1.15-1.36		1.72	1.56-1.87	
**Smoking**[1]						
Natural Indirect Effect	1.02	0.98-1.07	9%	1.04	0.99-1.09	8%
Natural Direct Effect	1.28	1.17-1.39		1.76	1.60-1.92	
Total Effect	1.31	1.21-1.40		1.82	1.67-1.97	

[1] Adjusted for cohabitation, age, country, sex and wave

Supplementary table 3 Total, natural direct and natural indirect effect of education on less than good self-rated health (n=15,028) for low vs. middle educated group and low vs. high educated group with working conditions as mediators

	Low versus middle socioeconomic position			Low versus high socioeconomic position		
	Relative Risk	95% CI	Proportion Mediated	Relative Risk	95% CI	Proportion Mediated
**Physically demanding job**[1]						
Natural Indirect Effect	1.07	1.01-1.12	27%	1.10	1.02-1.19	21%
Natural Direct Effect	1.22	1.12-1.33		1.65	1.47-1.82	
**Lack of job control**[1]						
Natural Indirect Effect	1.04	0.99-1.09	17%	1.09	1.02-1.16	18%
Natural Direct Effect	1.25	1.14-1.36		1.67	1.50-1.84	
**Lack of rewards**[1]						
Natural Indirect Effect	1.02	0.97-1.06	8%	1.04	0.98-1.09	8%
Natural Direct Effect	1.28	1.17-1.39		1.76	1.59-1.92	
Total Effect	1.31	1.21-1.40		1.82	1.67-1.97	

[1] Adjusted for cohabitation, age, country, sex and wave

Supplementary table 4 Total, natural direct and natural indirect effect of education on less than good self-rated health (n=15,028) for low vs. middle educated group and low vs. high educated group with health behaviors and working conditions as mediators

	Low versus middle socioeconomic position			Low versus high socioeconomic position		
	Relative Risk	95% CI	Proportion Mediated	Relative Risk	95% CI	Proportion Mediated
**Working conditions**[1]						
Natural Indirect Effect	1.09	1.03-1.15	35%	1.16	1.06-1.26	31%
Natural Direct Effect	1.20	1.09-1.31		1.57	1.39-1.75	
**Health behaviours**[1]						
Natural Indirect Effect	1.07	1.02-1.12	27%	1.14	1.07-1.21	27%
Natural Direct Effect	1.22	1.12-1.33		1.60	1.44-1.76	
**Health behaviors & working conditions**[1]						
Natural Indirect Effect	1.14	1.08-1.21	53%	1.32	1.20-1.45	54%
Natural Direct Effect	1.14	1.04-1.25		1.38	1.21-1.54	
Total Effect	1.31	1.21-1.40		1.82	1.67-1.97	

[1] Adjusted for cohabitation, age, country, sex and wave

Supplementary table 5: Total, natural direct and natural indirect effect of education on less than good self-rated health (n=15,028) for low vs. middle educated group and low vs. high educated group with health behaviors and working conditions as mediators, traditional mediation analysis

	**Low versus middle socioeconomic position**			**Low versus high socioeconomic position**		
	Relative Risk	95% CI	Proportion Mediated	Relative Risk	95% CI	Proportion Mediated
**Working conditions[1]**						
Natural Indirect Effect	1.05	1.03-1.06	32%	1.14	1.11-1.16	37%
Natural Direct Effect	1.11	1.04-1.19		1.30	1.20-1.41	
**Health behaviours[1]**						
Natural Indirect Effect	1.03	1.02-1.04	17%	1.06	1.05-1.08	18%
Natural Direct Effect	1.14	1.06-1.22		1.39	1.29-1.50	
**Health behaviors & working conditions[1]**						
Natural Indirect Effect	1.07	1.05-1.09	46%	1.19	1.16-1.23	50%
Natural Direct Effect	1.09	1.02-1.17		1.24	1.14-1.34	
Total Effect	1.17	1.09-1.25		1.48	1.37-1.60	

[1] Adjusted for self-rated health at baseline, cohabitation, age, country, sex and wave

Supplementary table 6: Total, natural direct and natural indirect effect of education on less than good self-rated health for low vs. high educated group with health behaviors as mediators, regional analysis

	Northern (n=2,187)[2]			Western (n=6,990)[2]			Southern (n=2,344)[2]			Eastern (n=2,939)[2]		
	Relative Risk	95% CI	PM	Relative Risk	95% CI	PM	Relative Risk	95% CI	PM	Relative Risk	95% CI	PM
**BMI[1]**												
Natural Indirect Effect	0.98	0.81-1.16	-4%	1.11	1.02-1.20	18 %	0.98	0.92-1.05	-4%	1.05	0.98-1.13	14 %
Natural Direct Effect	1.82	1.25-2.38		2.00	1.67-2.33		1.79	1.33-2.25		1.48	1.27-1.68	
**Alcohol[1]**												
Natural Indirect Effect	0.97	0.78-1.16	-8%	1.08	1.00-1.17	14 %	1.02	0.96-1.07	3%	1.05	0.97-1.14	14 %
Natural Direct Effect	1.85	1.26-2.44		2.05	1.72-2.39		1.74	1.30-2.18		1.48	1.27-1.69	
**Smoking[1]**												
Natural Indirect Effect	1.05	0.86-1.24	10%	1.01	0.94-1.07	1%	0.99	0.94-1.05	-1%	1.10	0.99-1.20	24 %
Natural Direct Effect	1.71	1.16-2.25		2.21	1.87-2.56		1.77	1.32-2.22		1.42	1.20-1.64	
**All health behaviors[1]**												
Natural Indirect Effect	1.20	0.90-1.49	37%	1.20	1.09-1.32	31 %	1.01	0.93-1.09	3%	1.11	0.99-1.23	27 %
Natural Direct Effect	1.49	0.96-2.02		1.85	1.52-2.18		1.74	1.28-2.19		1.41	1.18-1.63	
Total Effect	1.79	1.33-2.25		2.23	1.90-2.55		1.76	1.33-2.20		1.56	1.37-1.75	

[1] Adjusted for cohabitation, age, country, sex and wave

[2] Northern Europe (Sweden, Denmark), Western and Central Europe (Belgium, The Netherlands, France, Germany, Switzerland, Austria), Southern Europe (Greece, Spain, Italy), and Eastern Europe (Czech Republic, Poland and Estonia) – Ireland, Slovenia left outside of region analysis

Supplementary table 7: Total, natural direct and natural indirect effect of education on less than good self-rated health for low vs. high educated group with working conditions as mediators, regional analysis

	Northern (n=2,187)[2]			Western (n=6,990)[2]			Southern (n=2,344)[2]			Eastern (n=2,939)[2]		
	Relative Risk	95% CI	PM	Relative Risk	95% CI	PM	Relative Risk	95% CI	PM	Relative Risk	95% CI	PM
**Physically demanding job[1]**												
Natural Indirect Effect	0.97	0.73-1.21	-6%	1.14	1.03-1.25	22%	1.19	1.04-1.34	36%	1.05	0.92-1.19	14%
Natural Direct Effect	1.84	1.20-2.47		1.96	1.62-2.29		1.49	1.07-1.90		1.48	1.23-1.73	
**Lack of job control[1]**												
Natural Indirect Effect	1.02	0.83-1.20	3%	1.09	0.99-1.20	16%	1.06	0.97-1.16	14%	1.09	0.98-1.21	24%
Natural Direct Effect	1.76	1.21-2.31		2.04	1.68-2.39		1.66	1.22-2.10		1.42	1.19-1.66	
**Lack of rewards[1]**												
Natural Indirect Effect	0.94	0.78-1.09	-15%	1.01	0.94-1.08	2%	1.06	0.99-1.14	13%	1.10	1.00-1.19	24%
Natural Direct Effect	1.90	1.32-2.49		2.20	1.86-2.55		1.66	1.23-2.09		1.42	1.21-1.63	
**All working conditions[1]**												
Natural Indirect Effect	0.98	0.71-1.25	-4%	1.25	1.10-1.39	36%	1.32	1.11-1.54	56%	1.05	0.91-1.20	14%
Natural Direct Effect	1.82	1.15-2.49		1.78	1.45-2.11		1.33	0.94-1.73		1.48	1.22-1.74	
Total Effect	1.79	1.33-2.25		2.23	1.90-2.55		1.76	1.33-2.20		1.56	1.37-1.75	

[1] Adjusted for cohabitation, age, country, sex and wave

[2]Northern Europe (Sweden, Denmark), Western and Central Europe (Belgium, The Netherlands, France, Germany, Switzerland, Austria), Southern Europe (Greece, Spain, Italy), and Eastern Europe (Czech Republic, Poland and Estonia) – Ireland, Slovenia left outside of region analysis

Supplementary table 8: Regional characteristics for mediators among imputed dataset of employed individuals

Northern region	Low educational level n=435 (%)	Intermediate educational level n=672 (%)	High educational level n=1,080 (%)
**Health behaviors**			
BMI			
Normal	40	40	54
Overweight	45	42	36
Obese	16	18	10
Smoking			
Non-smoking	31	37	46
Current	31	27	19
Former	39	36	35
Alcohol consumption			
Hardly ever/never	18	13	11
1–2 days per week	66	52	52
3–4 days per week	7	15	19
5 or more days per week	9	20	18
**Working conditions**			
Physically demanding job	61	47	31
Lack of job control	33	27	16
Lack of job rewards	36	39	31

Southern region	Low educational level n=1,146 (%)	Intermediate educational level n=624 (%)	High educational level n=574 (%)
**Health behaviors**			
BMI			
Normal	30	36	45
Overweight	49	52	43
Obese	21	12	12
Smoking			
Non-smoking	40	42	39
Current	35	35	30
Former	25	24	30
Alcohol consumption			
Hardly ever/never	38	35	33
1–2 days per week	22	29	33
3–4 days per week	7	7	9
5 or more days per week	33	28	24
**Working conditions**			
Physically demanding job	72	48	33
Lack of job control	32	21	12
Lack of rewards	40	32	24

Supplementary table 9: Multivariate odds ratios of the association between baseline work characteristics and self-rated health at follow-up after adjustment for demographic factors (education, cohabitation, gender, age) and self-rated health at baseline.

	**Self-rated health at t1**
Work characteristics*	
High physical work demands	1.36 (1.24-1.50)
Low job control	1.42 (1.29-1.57)
Low job rewards	1.34 (1.22-1.48)
Control variables*	
Education	
High education	1.00
Mid education	1.28 (1.15-1.44)
Low education	1.50 (1.32-1.71)
Self-rated health at baseline	
Good or better	1.00
Poor or less	8.48 (7.66-9.38)
Gender	
Male	1.00
Female	1.00 (0.91-1.09)
Age	
50-54 years old	1.00
55-59 years old	1.14 (1.03-1.26)
60-64 years old	1.20 (1.05-1.38)
Cohabitation	
Single	1.00
Married or partnership	0.91 (0.82-1.02)

* also controlled for country and wave
